# Infliximab‐induced autoimmune hepatitis requiring liver transplantation

**DOI:** 10.1002/ccr3.2456

**Published:** 2019-09-27

**Authors:** Frederick Wong, Bashaar Al Ibrahim, Joanna Walsh, Karim Qumosani

**Affiliations:** ^1^ Division of Gastroenterology Department of Internal Medicine Western University London Ontario Canada; ^2^ Department of Medicine King Faisal Special Hospital and Research Centre Riyadh Saudi Arabia; ^3^ Department of Pathology and Laboratory Medicine Western University London Ontario Canada

**Keywords:** autoimmune hepatitis, Crohn's disease, infliximab, liver transplantation

## Abstract

Autoimmune hepatitis is an infrequent but significant side effect of infliximab treatment. Diagnosis of autoimmune hepatitis is based on clinical, laboratory, and histological findings. Initial treatment involves cessation of infliximab and trial of prednisone. We present a rare case of infliximab‐induced autoimmune hepatitis leading to liver failure requiring transplantation.

## Graphical abstract

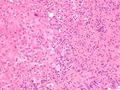

## 1 INTRODUCTION

Infliximab, a monoclonal antibody that inhibits tumor necrosis factor α (TNF‐α), is commonly used in the treatment of inflammatory conditions such as Crohn's disease and rheumatoid arthritis. A rare but significant side effect of infliximab treatment is autoimmune hepatitis. Other autoimmune conditions induced by infliximab include systemic lupus erythematosus, psoriasis, and vasculitis.^1^, ^2^ Autoimmune hepatitis following infliximab treatment has been previously described in the treatment of patients with Crohn's disease,^3^, ^4^, ^5^, ^6^, ^7^ ulcerative colitis,^8^, ^9^ rheumatoid arthritis,^10^ psoriasis,^11^, ^12^, ^13^ ankylosing spondylitis,^14^ and psoriatic arthritis.^15^ Most of these cases resolved with cessation of infliximab, but some required additional steroid therapy. Here, we present the second case reported in the literature of a patient with Crohn's disease treated with infliximab that developed autoimmune hepatitis requiring liver transplantation.^16^

## 2 CASE

We present a 69‐year‐old woman with a history of fistulizing Crohn's disease for over 13 years. She had multiple previous small bowel surgeries including resections and fistulectomy. The patient was started on infliximab because of the development of new fistulas. Her past medical history included hypertension, osteoarthritis, gastroesophageal reflux disease, and sigmoid diverticular disease. Her medications were amlodipine, rabeprazole, vitamin D, and furosemide. She had no significant social history and rarely consumed alcohol.

Approximately 3 months into infliximab treatment after three separate doses of 5mg/kg of infliximab, she was found to be jaundiced on physical examination prior to her fourth dose at the infusion clinic. Blood work confirmed an elevated total bilirubin 392.5 µmol/L, which was mainly direct > 236.0 µmol/L. Liver enzymes were also significantly elevated (Table 1) and were previously documented as normal 6 months prior to her presentation. Her synthetic liver function was abnormal with a low albumin and elevated INR. Her autoimmune markers showed an elevated IgG and slightly elevated IgA. Antismooth muscle antibodies (ASMAs) were positive, antinuclear antibodies (ANAs) were positive, and antimitochondrial antibodies (AMAs) were negative. Hepatitis B and C serology, ferritin, ceruloplasmin, and alpha‐1‐antitrypsin were negative.

**Table 1:** Laboratory data on presentation

Liver Enzymes	
ALT (<33 U/L)	647
AST (<33 U/L)	1115
GGT (≤31 U/L)	400
ALP (35‐104 U/L)	256
Synthetic liver function	
Total bilirubin (3.4‐17.1 µmol/L)	392.5
Direct bilirubin (0.0‐5.0 µmol/L)	>236.0
INR (0.9‐1.1)	3.9
PTT (23‐32 s)	42
Albumin (35‐52 g/L)	27
Immunology	
Immunoglobulin G (6.4‐13.8 g/L)	27.9
Immunoglobulin A (0.9‐4.6 g/L)	6.0
Immunoglobulin M (0.6‐2.7 g/L)	1.4
ANA (Neg < 1 in 40)	<1:80
AMA (Neg < 1 in 20)	<1:20
ASMA (Neg < 1 in 20)	1:40
Infectious serology	
Hepatitis B surface antigen	Nonreactive
Hepatitis B core antibody	Nonreactive
Hepatitis C antibody	Nonreactive
Hematology	
Hemoglobin (115‐160 g/L)	107
Leukocytes (4.0‐10.0 × 10^9^ g/L)	7.6
Platelets (150‐400 × 10^9^ g/L)	269

Abbreviations: ALP, alkaline phosphatase; ALT, alanine aminotransferase; AMA, antimitochondrial antibody; ANA, antinuclear antibody; ASMA, antismooth muscle antibody; AST, aspartate aminotransferase; GGT, gamma‐glutamyl transferase; INR, international normalized ratio; PTT, partial thromboplastin time.

**Figure 1:**
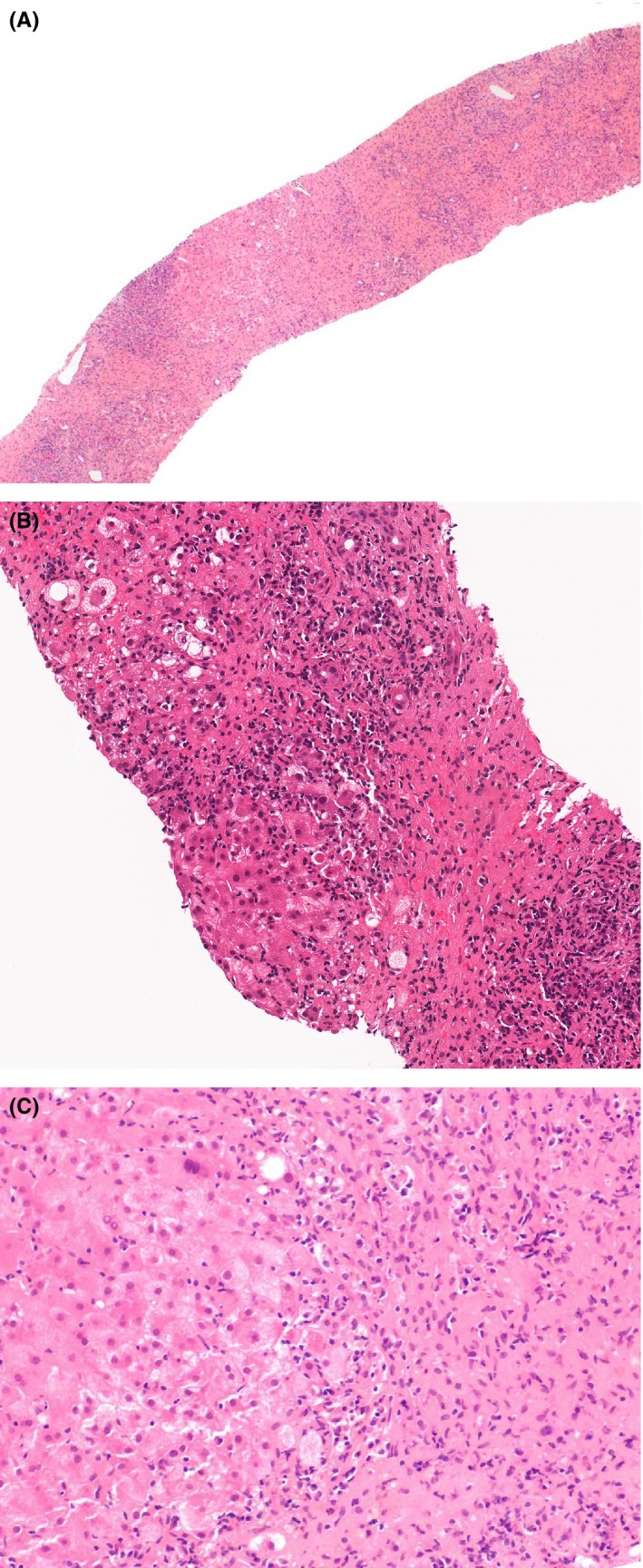
Liver biopsy revealing (A) acute hepatitis with parenchymal necrosis (H&E, 4×), B,Hepatocyte injury and apoptosis with dense plasma cell infiltrate (H&E, 20×). C, Interface hepatitis with plasma cells (H&E, 10×)

Ultrasound of the liver showed nonspecific diffuse heterogeneous coarse hepatic parenchyma with no focal lesions and no extra hepatic biliary obstruction. Magnetic resonance cholangiopancreatography was done and showed no evidence of intrahepatic or extrahepatic duct dilatation. Liver biopsy revealed extensive multiacinar hepatocyte necrosis with areas of surviving hepatocytes, a periportal and lobular infiltrate of lymphocytes and plasma cells, and interface hepatitis, morphologically consistent with autoimmune hepatitis. There was no evidence of chronic fibrosis (Figure 1).

**Figure 2:**
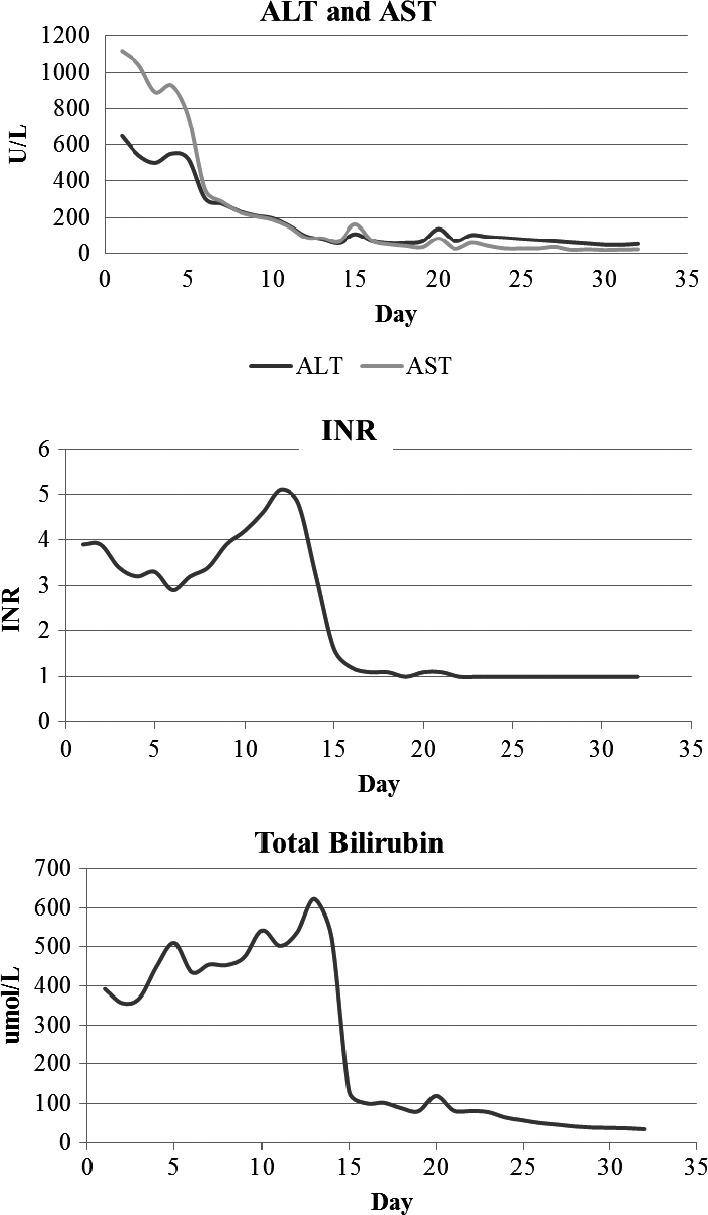
Clinical course since admission

The patient was admitted to the hospital for further evaluation and liver transplant assessment. She was started on prednisone 30 mg daily; however, there was no improvement. During her hospitalization, her liver enzymes continued to increase (Figure 2). Her synthetic liver function also continued to deteriorate. The patient developed acute kidney injury which was attributed to hepatorenal syndrome. Her condition continued to worsen, and she developed hepatic encephalopathy.

**Figure 3:**
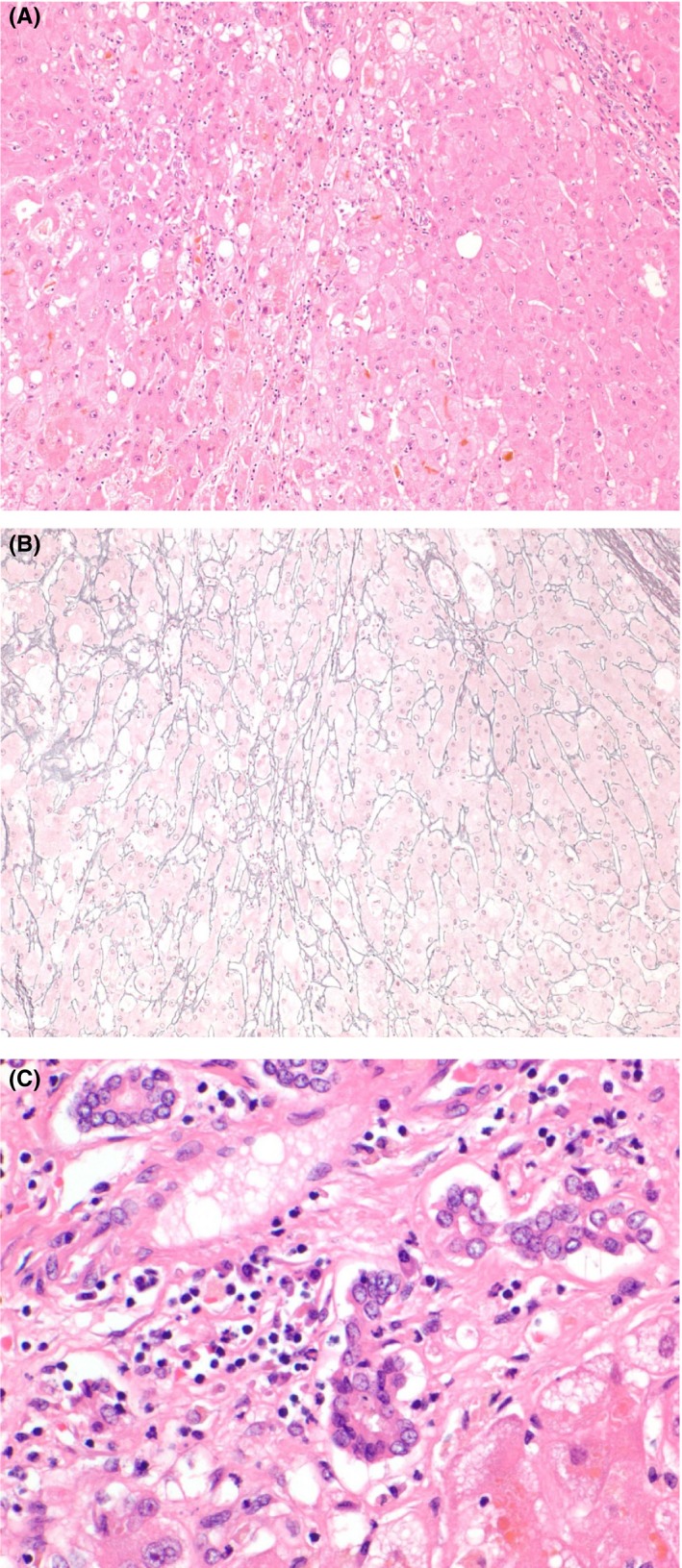
Liver explant (A) parenchymal collapse (H&E, 10×). B, Parenchymal collapse (reticulin, 10×).C, Portal plasma cells (H&E, 20×)

The liver transplant team evaluated the patient and deemed her a good candidate for liver transplantation. Two weeks after her admission, the patient underwent liver transplantation. Further examination of the explanted liver confirmed the findings of the initial biopsy with parenchymal collapse and the presence of portal plasma cells (Figure 3). The patient was discharged home 2 weeks after her transplant and was doing well at the 3‐, 6‐, and 12‐month visits after transplantation. Repeated antibody panel showed seroconversion of ASMA and ANA from positive to negative 3 months after the liver transplant. Colonoscopy one year following the liver transplant revealed mild recurrent Crohn's disease. Subsequent colonoscopy performed two and a half years postliver transplantation showed recurrent Crohn's with stricturing and moderate neoterminal ileitis. Ustekinumab was offered at this time due to prior infliximab treatment leading to autoimmune hepatitis.

## 3 DISCUSSION

We present a rare case of infliximab‐induced autoimmune hepatitis requiring liver transplantation here. There are only a few cases of liver transplantation following infliximab treatment previously described in the literature.^16^, ^17^, ^18^, ^19^ However, several of these patients had factors that could have contributed to the transplantation including prior cirrhosis and hepatitis infection.^17^, ^18^ One case involved a 39‐year‐old female patient with rheumatoid arthritis who was treated with infliximab and later required a liver transplantation.^17^ Liver biopsy revealed underlying cirrhosis in this patient, possibly contributing to the need for transplantation. Another case is a 28‐year‐old adult‐onset Still's patient with prior hepatitis B infection treated with infliximab that developed fulminant hepatitis requiring liver transplantation.^18^ The patient was positive for hepatitis B surface antigen and antihepatitis B e‐antigen antibodies at the time of presentation, but negative for hepatitis B virus DNA. The underlying hepatitis B may have possibly contributed to the need for liver transplant. In fact, multiple cases of hepatitis B reactivation following infliximab treatment have been previously reported^20^, ^21^ with one case even leading to fulminant liver failure.^22^ Another case of fulminant hepatic failure in a patient with Crohn's disease treated with infliximab has been reported; however, the liver failure was attributed to herpes simplex virus reactivation.^23^ Thus, our case of autoimmune hepatitis following infliximab treatment requiring transplantation is unique in that our patient did not have prior cirrhosis or hepatic infection that would have contributed to needing a liver transplantation.

The diagnosis of autoimmune hepatitis is made based on clinical, laboratory, and histological findings. The simplified autoimmune hepatitis score is a useful tool developed by the international autoimmune hepatitis group in 2008 based on ANA levels (<1:40 = 1 point, <1:80 = 2 points), IgG (upper normal limit = 1 point, >1.10 times normal limit = 2 points), liver histology (compatible with autoimmune hepatitis = 1 point, typical for autoimmune hepatitis = 2 points), and absence of viral hepatitis (yes = 2 points, no = 0 point) to calculate the probability of autoimmune hepatitis. A score of greater or equal to 7 is definite autoimmune hepatitis, and a score of greater or equal to 6 is probable autoimmune hepatitis. Our patient in this case report received a score of 7 with ANA of < 1:80, IgG > 1.10 times normal limit, histology compatible with autoimmune hepatitis, and absence of viral hepatitis.^24^

Elevated autoimmune antibodies, histological findings consistent with autoimmune hepatitis, and lack of other causal factors strongly suggest that this patient presented with infliximab‐induced autoimmune hepatitis. Additionally, the findings were felt to be more consistent with autoimmune hepatitis than drug‐induced liver injury. Liver biopsy results for autoimmune hepatitis classically reveal interface hepatitis, focal necrosis, portal inflammation, and plasma cells which were present in this case.^25^ Meanwhile, findings with drug‐induced liver injury are more likely to show portal neutrophils and hepatocellular cholestasis.^26^ There is also the possibility that autoimmune hepatitis developed independently of infliximab treatment since autoimmune hepatitis is often associated with other autoimmune conditions including celiac disease and rheumatoid arthritis.^27^ This is unlikely in this case given the development of autoimmune hepatitis immediately after infliximab treatment and the fact that normal liver enzymes were detected several months prior to starting infliximab treatment. It is also crucial to rule out other potential causes of hepatitis including alcohol use, viral infection, and hepatotoxic medications which were all not present in this case.

Other TNF‐α inhibitors including etanercept^28^ and adalimumab^29^, ^30^ have reportedly also resulted in autoimmune hepatitis. Some of the mechanisms proposed include underlying genetic susceptibility, a selective effect of T helper cells and immune complex formation, and the induction of an immune system imbalance from cytokine blockade.^30^ It is likely that the autoimmune response to TNF‐α inhibitors is individualized since there have also been reports of patients who developed autoimmune hepatitis following infliximab treatment that did not exhibit any liver dysfunction after switching to etanercept^10^ and adalimumab.^31^ Proposed monitoring for liver impairment during TNF‐α inhibitor treatment includes regular liver function tests every two to eight weeks with a referral to a hepatologist recommended when ALT is >3 times the upper limit of normal or when there is an increase in bilirubin or onset of jaundice.^32^

In conclusion, clinicians must be aware of the rare possibility of developing autoimmune hepatitis following infliximab treatment. Treatment involves discontinuation of infliximab and trial of steroids. In severe cases with liver failure, transplantation may be necessary.

## CONFLICT OF INTEREST

The authors do not have any conflicts of interest to declare.

## AUTHOR CONTRIBUTIONS

Frederick Wong: involved in drafting and finalizing the manuscript. Bashaar Al Ibrahim: involved with collecting the data and drafting the manuscript. Joanna Walsh: involved with preparing the descriptions for the figures and editing the manuscript. Karim Qumosani: involved with editing the manuscript.

## CONSENT

Informed consent was obtained.
